# Comparison of Structural and Physicochemical Characteristics of Skin Collagen from Chum Salmon (Cold-Water Fish) and Nile Tilapia (Warm-Water Fish)

**DOI:** 10.3390/foods13081213

**Published:** 2024-04-16

**Authors:** Yan Zheng, Yushuang Li, Cong Ke, Xiyuan Gao, Zhiyu Liu, Junde Chen

**Affiliations:** 1 Technical Innovation Center for Utilization of Marine Biological Resources, Third Institute of Oceanography, Ministry of Natural Resources, Xiamen 361005, China; zhengyan@tio.org.cn (Y.Z.); liyushuang@tio.org.cn (Y.L.); 13172508013@163.com (C.K.); gxygxy18649687417@163.com (X.G.); 2 Fisheries Research Institute of Fujian, Key Laboratory of Cultivation and High-Value Utilization of Marine Organisms in Fujian, Xiamen 361021, China

**Keywords:** chum salmon skin, Nile tilapia skin, collagen, structure, rheology properties, functional properties

## Abstract

This study compared collagens from cold-water and warm-water fish for their structural, rheological, and functional properties, and explored their potential applications, aiming to realize the high-value utilization of marine biological resources. To this end, chum salmon skin collagen (CSSC) and Nile tilapia skin collagen (NTSC) were both successfully extracted. Collagens from the two species had different primary and secondary structures, with NTSC having a higher molecular weight, imino acid content, and α-helices and β-turns content. The denaturation temperatures were 12.01 °C for CSSC and 31.31 °C for NTSC. CSSC was dominated by viscous behavior and its structure varied with temperature, while NTSC was dominated by elastic behavior and its structure remained stable with temperature. Both collagens had good oil holding capacity, foaming capacity, and emulsifying activity, but NTSC had better water holding capacity and foaming and emulsifying stability. Their different properties make CSSC more suitable for the preservation of frozen and chilled foods and the production of sparkling beverages, and give NTSC greater potential in biofunctional materials and solid food processing.

## 1. Introduction

Collagen is a structural protein of the extracellular matrices of animal tissues characterized by high biodegradability, biocompatibility, and low antigenicity [1]. Collagen is widely used in food processing, pharmaceutical, cosmetic, and biomedical applications [2]. The global collagen market was valued at USD 4.7 billion in 2020 and is estimated to reach USD 7 billion by 2027 [3]. Traditionally, the main sources of commercial collagen have been pigs and cattle, but the use of collagen from mammalian origin has been restricted due to religious beliefs and the potential risk of spreading zoonotic diseases [4]. Fish-derived collagen has become a hot research topic, as it is considered to be a safer and more attractive alternative than collagen from terrestrial animal sources [5]. Li et al. [6] isolated and characterized collagen from eel skin to investigate its thermal denaturation, structure, physicochemical properties, and allergenicity. Atef et al. [2] investigated the structural and biochemical properties of collagen from sturgeon fish skin. Tang et al. [7] successfully extracted collagen from the skin of tilapia, grass carp, and silver carp and evaluated the physicochemical properties of each.

While collagen has been extracted from various fish species by researchers, there has been limited research on fish collagen in different habitat water temperatures. As biochemical characteristics of collagens are sensitive to changes in animal habitat temperature, collagens in different water temperatures (warm or cold water) undergo corresponding changes in structure, function, and biochemical characteristics [8]. There is a lack of studies comparing the structural, physicochemical, rheological, and functional properties of fish collagen from different habitat water temperatures and exploring their applications. It is necessary to understand the influence of habitat water temperatures on the structural, physicochemical, and application properties of collagen in order to realize collagen’s potential in industrial production and to achieve optimal application of collagen.

Chum salmon (*Oncorhynchus keta*), a cold-water fish that matures in the ocean and coastal streams, with an optimum water temperature of 8–12 °C, is a commercially important fishery resource in North Pacific nations [9]. The average annual catch in 2022 of the major fishing nations in the North Pacific (Japan, USA, Canada, and Russia) was around 212,000 tons [10]. The salmon processing industry primarily utilizes fish flesh and roe. However, the skin of the fish is an underutilized byproduct of the processing process and contains a significant amount of collagen, making it an important resource [11]. Nile Tilapia (*Oreochromis niloticus*), a warm-water species with an optimum water temperature range of 25–30 °C, is an economically important farmed fish [12]. According to the China Fishery Statistical Yearbook, China’s freshwater tilapia production in 2022 was about 1.74 million tons, and the corresponding amount of aquaculture processing was about 540,000 tons. Tilapia processing produces a large amount of fish skin that is rich in collagen [13]. Using fish skin as raw materials for production of fish collagen can realize the high-value utilization of low-value fish processing products. Additionally, it can solve the environmental pollution problem caused by the large amount of byproducts produced in fish processing [14,15]. The effective utilization of fish processing byproducts can achieve the sustainable use of fish resources and promote the development of the circular economy in fish processing industry.

Therefore, the aim of this study was to extract collagen from the skin of a cold-water fish, chum salmon, and a warm-water fish, Nile tilapia, and to compare the structure, thermal stability, rheological properties, and functional properties of the two types of collagen to explore the potential applications. This study could thus provide reference data for the processing and industrial application of fish skin collagen from different habitat water temperature and could serve as a guide to the development of fish skin byproducts for high-value application.

## 2. Materials and Methods

### 2.1. Materials

Frozen fish skins of chum salmon (*Oncorhynchus keta*) and Nile tilapia (*Oreochromis niloticus*) were obtained from Beihai Quality Aquatic Products Co., Ltd. (Beihai, China). The skins obtained were stored at freezing temperature (−20 °C) until use. Rat tail type I collagen standard (C7661) was purchased from Sigma Chemical Company (St. Louis, MO, USA), and protein marker (26634) was purchased from Thermo Fisher Scientific Baltics (Vilnius, Lithuania). All chemical reagents used were of analytical grade.

### 2.2. Preparation of Collagen from Fish Skin

Collagen was prepared by referring to the method of Chen et al. [16] with slight modification. The fat tissue and scales remaining on the fish skin were removed, and the skin was washed with distilled water. The pretreated fish skin was dissolved in 0.5 M acetic acid at a solid/solvent ratio of 1:30 (*w*/*v*) and stirred overnight (EUROSTAR 20 digital, IKA, Burladingen, Germany). The solutions were centrifuged (Avanti J-26 XP, Beckman, South Kraemer Boulevard Brea, CA, USA) with a rotor (JA-10) at 8875× *g*, 4 °C for 30 min to remove the precipitate. The supernatant was added to 4% (*w*/*v*) NaCl, stirred thoroughly for 0.5 h, left to stand for 0.5 h, and the precipitate was retained by centrifugation at 8875× *g* for 30 min at 4 °C. The precipitate was redissolved in 1:15 (*w*/*v*) of 0.5 M acetic acid and stirred overnight. Completely dissolved samples were dialyzed in dialysis bags (MD 77 MM, Viskase, Darien, IL, USA), first in 0.1 M acetic acid for 36 h, and then in distilled water for 36 h. Finally, the dialyzed collagen solutions were lyophilized (Telstar, LyoBeta-25, Terrassa, Spain). Each of the above steps was operated at 4 °C, and the samples obtained were stored at −20 °C. In addition, the moisture content of the fish skin was measured, the fish skin was weighed and placed in a 105 °C drying oven (DHG-9023A, Shanghai, China), dried for 4 h and cooled for 0.5 h and weighed again.

The moisture content of fish skin was calculated using the following formula:

(1) $$X\left(\%\right)=\frac{M_{0}{-M}_{1}}{M_{0}}\times 100,$$

where *M*_0_ is the weight of before drying (g) and *M*_1_ is the weight after drying (g).

The yield of fish skin collagen was calculated using the following equation:

(2) $$Yield\left(\%\right)=\frac{M_{2}}{M\times \left(1-X\right)}\times 100,$$

where *M*_2_ is the weight of lyophilised collagen (g), X is the moisture content of fish skin (%), and *M* is the weight of the raw material (g).

### 2.3. Structural Analysis

#### 2.3.1. Sodium Dodecyl Sulphate-Polyacrylamide Gel Electrophoresis (SDS-PAGE)

The collagen was prepared as a 1 mg/mL sample solution, mixed with the 4× Laemmli Sample Buffer (1610747, Bio-Rad Laboratories, Hercules, CA, USA), and then heated at 100 °C for 3 min. An 8% separating gel and 3% stacking gel were prepared. A protein marker (26634) was used to estimate the molecular weight of the samples, and rat tail type I collagen was used as a reference. The samples were electrophoresed for 65 min at 110 V, 60 mA using an electrophoresis system (Bio-Rad Laboratories, Hercules, CA, USA). The gels were fixed with 50% (*v*/*v*) ethanol and 10% acetic acid for 30 min, stained with 0.125% (*w*/*v*) Coomassie Brilliant Blue, 50% (*v*/*v*) ethanol, and 10% acetic acid for 30 min, and decolorized using 50% methanol and 10% acetic acid for 20 min. Finally, the protein molecular weights were estimated using Quantity One software (VERSION 4.6.0, Bio-Rad Laboratories, Hercules, CA, USA), and the bands were analyzed using Image J software (VERSION 1.8.0, National Institute of Mental Health, Bethesda, MD, USA).

#### 2.3.2. Protein Sequence Analysis

The determination of collagen sequences used the LC-MS/MS method described by Chen et al. [16] with slight modifications. Collagen was dissolved in distilled water for electrophoresis, and the obtained α1 and α2 bands were cut and subjected to enzymatic hydrolysis pretreatment. The concentrated and dried samples were employed for online LC-MS analysis, where the liquid phase was an Easy-nLC1200 liquid phase system (Thermo Scientific, Waltham, MA, USA), and mass spectrometry employed a QExactive system (Thermo Scientific, Waltham, MA, USA). The original files were imported into the Mascot software (VERSION 2.3.02, Matrix Science Inc., London, UK) search engine for protein identification. The search parameters were as follows: the database was NCBI; the enzyme setting was trypsin; the maximum allowed missed cut site was 1; and the protein score C.I.% was greater than 95% for successful identification.

#### 2.3.3. Ultraviolet (UV) Absorption Spectra

A solution of 1 mg/mL collagen was prepared with a 0.5 M acetic acid solution, and 0.5 M acetic acid was used as the blank control. The UV absorption value of the sample solution was measured using a UV–Vis spectrophotometer (UV-1780 SHIMADZU, Kyoto, Japan) at a wavelength range of 220–400 nm with a wavelength interval of 1 nm and a high scanning speed.

#### 2.3.4. Fourier Transform Infrared (FTIR) Spectra

The sample was mixed with potassium bromide at a ratio of 1:100, ground, and pressed until a translucent sample flake was formed, with potassium bromide as the background flake. The background channel and sample channel spectrum were measured using a Bruker FTIR spectrophotometer (VERTEX 70, Bruker, Bremen, Germany) with the following parameters: scanning range 4000–400 cm^−1^, resolution 4 cm^−1^.

#### 2.3.5. FTIR Spectral Curve Fitting

The second-order FTIR spectra were analyzed using OMNIC software (VERSION 8.2, Thermo Nicolet, Waltham, MA, USA), and the spectra were analyzed by fitting a Gaussian curve using PeakFit software (VERSION 4.12, SeaSolve Software Inc., Shelton, CT, USA). The fitting was performed to minimize the standard error with R^2^ > 0.999 to verify the quality of the Gaussian curve fit. The percentage of each band area to the area of the 1700–1600 cm^−1^ region was determined as the content of the corresponding protein secondary structure.

#### 2.3.6. X-ray Diffraction (XRD)

The collagen samples were measured in an X-ray diffraction system (X’Pert Pro XRD, PANalytical, Almelo, The Netherlands) with a scanning range of 5–90° (2*θ*) and a scanning speed of 10°/min. The Bragg equation was used to calculate the d values as follows:

(3) $$d\left(Å\right)=\frac{\lambda }{2sin\theta },$$

where *λ* (1.54°) is the X-ray wavelength and *θ* is the Bragg diffraction angle.

### 2.4. Determination of Denaturation Temperature (Td)

The thermal stability of collagen was analyzed via Td, with reference to Gao et al. [17]. The collagen solution was prepared with 0.5 M acetic acid at a concentration of 30 mg/mL, and the change in collagen viscosity at different temperatures (0–45 °C) was measured using a rheometer (MCR 302, Anton Paar, Graz, Austria) with a steel cone/plate (2° cone angle, 25 mm cone diameter) at a temperature increase rate of 3 °C/min. The temperature at which the relative viscosity was 50% was used as the Td of the samples.

### 2.5. Rheological Properties

#### 2.5.1. Dynamic Frequency Sweep Tests

The rheological experiments referred to the method of Li et al. [18] with slight modification. The collagen was dissolved in 0.5 M acetic acid solution, and dynamic frequency scanning tests were performed at 10 °C for different concentrations (10, 20, 30, and 40 mg/mL) and at 30 mg/mL for different temperatures (5, 15, 25, 35, and 45 °C). A rheometer (MCR 302, Anton Paar, Austria) was used with a steel cone/plate (0.5° cone angle, 60 mm cone diameter) with a frequency scan range of 0.01–10 Hz with a constant strain of 30%.

#### 2.5.2. Steady Shear Tests

The collagen was dissolved in 0.5 M acetic acid solution, and steady-state shear tests were performed at 10 °C for different concentrations (10, 20, 30, and 40 mg/mL) and at 30 mg/mL for different temperatures (5, 15, 25, 35, and 45 °C). A rheometer (MCR 302, Anton Paar, Austria) with a steel cone/plate (0.5° cone angle, 60 mm cone diameter) was used, with a shear rate range of 0.1–100 Hz and a rotational mode. In addition, the Ostwald model was used to further describe the relationship between viscosity and shear rate [19]:

(4) $$\eta =K\gamma^{n-1},$$

where *η* is the shear viscosity (Pa·s); *K* is the consistency coefficient (Pa·s^n^); *γ* is the shear rate (s^−1^); and *n* is the flow behavior index.

### 2.6. Functional Properties

#### 2.6.1. Water Holding Capacity (WHC)

The determination of WHC referred to the experimental method of Chandi et al. [20] with slight modification. Collagen (0.2 g) was added to 10 mL of distilled water, shaken for 120 s, left at room temperature for 1 h, and centrifuged at 5000 r/min for 30 min. WHC was reported as the weight of absorbed water per unit weight of the sample (g/g).

#### 2.6.2. Oil Holding Capacity (OHC)

The determination of OHC referred to the experimental method of Chandi et al. [20] with slight modification. Collagen (0.2 g) was added to 10 mL of oil, shaken for 120 s, left for 1 h at room temperature, and centrifuged at 5000 r/min for 30 min. OHC was reported as the weight of absorbed oil per unit weight of the sample (g/g).

#### 2.6.3. Foaming Capacity (FC) and Foaming Stability (FS)

The determination of FC and FS referred to the experimental method of Çelik et al. [21] with slight modification. The collagen sample was dissolved in 0.5 M acetic acid solution to prepare a 0.3% (*w*/*v*) collagen solution. An amount of 25 mL of the collagen solution was placed in a 50 mL measuring cylinder and was homogenized at 23,000 r/min for 2 min (JS25, JUNRUI, Yangzhou, China); the volume was recorded immediately as *V*_1_. After standing for 30 min, the volume was recorded as *V*_2_. The FC and FS values of the samples were as follows:

(5) $$FC\left(\%\right)=\frac{V_{1}-V_{0}}{V_{0}}\times 100,$$

(6) $$FS\left(\%\right)=\frac{V_{2}-V_{0}}{V_{1}-V_{0}}\times 100,$$

where *V*_0_ is the volume before homogenization (mL); *V*_1_ is the volume after homogenization (mL); and *V*_2_ is the volume after 30 min (mL).

#### 2.6.4. Emulsifying Activity Index (EAI) and Emulsifying Stability Index (ESI)

The determination of EAI and ESI values referred to the experimental method of Çelik et al. [21] with slight modification. A 0.3% (*w*/*v*) collagen solution was prepared by dissolving the collagen sample in 0.5 M acetic acid solution. Then, 15 mL of collagen solution was added with 5 mL of oil, homogenised at 23,000 r/min for 60 s (JS25, JUNRUI, Yangzhou, China). A total of 100 μL of the bottom solution was transferred to a centrifuge tube at 0 min and 10 min, diluted 100 times with 0.1% SDS, and the absorbance was measured at 500 nm using 0.1% SDS as blank control. The EAI and ESI values of the samples were calculated as follows:

(7) $$EAI\left(m^{2}/g\right)=\frac{2\times 2.303\times A_{0}\times DF}{c\times \varphi \times 10000},$$

(8) $$ESI\left(min\right)=\frac{A_{0}\times \Delta t}{∆A}$$

where *A*_0_ is the absorbance at 0 min; DF is the dilution multiple; *c* is the collagen concentration; *φ* is the oil volume fraction (0.25); ∆*t* is 10 min; *A*_10_ is the absorbance after 10 min of homogenization; and ∆*A* is *A*_0_ − *A*_10_.

### 2.7. Statistical Analysis

All experiments were performed in triplicate, and the results were calculated as the average ± standard deviation using SPSS software (VERSION 17.0, IBM SPSS Statistics, Enningen, Germany).

## 3. Results and Discussion

### 3.1. Yield

Chum salmon skin collagen (CSSC) and Nile tilapia skin collagen (NTSC) yields (dry basis weight) were 34.25 ± 1.22% and 44.32 ± 0.60%, respectively. The yields of CSSC and NTSC were higher than the yields of skin collagen from red stingray (33.95 ± 0.7%) [22], yellowfin tuna (18.5 ± 1.55%) [23], and black carp (15.5%) [5], indicating that these two types of collagen had high application value. The higher yield of NTSC than CSSC might indicate that collagen from fish living in warm water environments was easier to extract than collagen from fish living in cold water environments.

### 3.2. Structural Analysis

#### 3.2.1. SDS-PAGE

**Figure 1:**
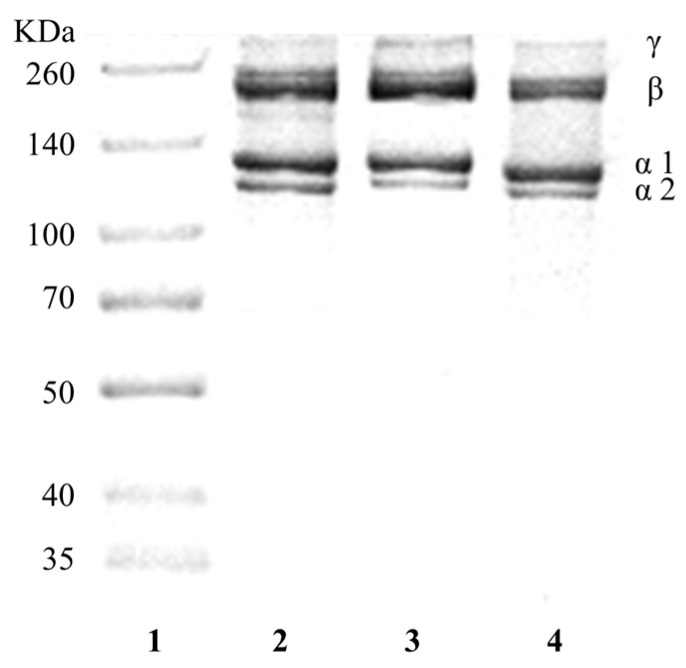
SDS-PAGE pattern of CSSC and NTSC. Lane 1: protein marker; Lane 2: rat tail type I collagen; Lane 3: NTSC; Lane 4: CSSC.

As shown in Figure 1, both CSSC and NTSC were composed of different α-chains (α1, α2) and both contained dimeric β-chains and trimeric γ-chains formed by intra- and intermolecular crosslinking of collagen molecules, similar to the rat tail type I collagen standard. Similar results were found in studies of type I fish skin collagen from sturgeon fish [2], skipjack tuna [24], and black carp [5]. The results of the grey scale analysis of Image J showed that the ratios of α1 to α2 of CSSC and NTSC were 2.06 and 2.25, respectively, which were close to 2. This was consistent with the structure of [α1(I)_2_α2(I)] of the rat tail type I collagen standard, suggesting that CSSC and NTSC were type I collagen. The γ- and β-chain contents of NTSC were higher than those of CSSC, indicating that the degrees of intra- and intermolecular crosslinking of NTSC were greater, and thus that its stability would be stronger than that of CSSC; this might be related to the fact that tilapia is a warm-water fish with a greater tolerance to higher temperatures. The molecular weights of collagen were calculated using Quantity One. The α1-chain molecular weight was 126 kDa, and the α2-chain molecular weight was 117 kDa for CSSC, while the α1-chain molecular weight was 130 kDa, and the α2-chain molecular weight was 121 kDa for NTSC.

#### 3.2.2. Protein Sequence Analysis

Collagen samples were digested with trypsin and sequenced using an LC-MS/MS system. The data were matched to the NCBI database using Mascot, and the matching proteins were identified by the primary and secondary fragment quality of the peptides (a match greater than 95% was considered reliable), and then the proteins were identified based on the protein score, the number of matching peptides, and the protein sequence coverage [22]. The results of the protein identification are shown in Table S1. The CSSCα1 match was for the *Oncorhynchus keta* type I collagen α1-chain with a score of 1996, a matching peptide count of 28, and a coverage of 30%; the CSSCα2 match was for the *Oncorhynchus keta* type I collagen α2-chain with a score of 1219, a matching peptide count of 28, and a coverage of 30%; the NTSCα1 match was for the *Oreochromis niloticus* type I collagen α1-chain with a score of 527, a matching peptide count of 18, and a coverage of 21%; the NTSCα2 match was for the *Oreochromis niloticus* type I collagen α2-chain with a score of 906, a matching peptide count of 22, and a coverage of 25%. Therefore, we could conclude that the prepared CSSCα1 and CSSCα2 were *Oncorhynchus keta* type I collagen and that the NTSCα1 and NTSCα2 were *Oreochromis niloticus* type I collagen. The amino acid sequences are shown in Figures S1 and S2.

Collagen has a macroscopic molecular structure consisting of a unique triple helix formed by three intertwined α-subunits, each containing a (Gly-X-Y)_n_ conserved sequence, where X and Y are usually occupied by Pro and Hyp [17]. Based on the amino acid sequences identified, the α1 and α2 chains of CSSC and NTSC comprised similar structures, both consisting of a signal peptide domain, a triple-helical domain, N-propeptide and C-propeptide domains, and N-telopeptide and C-telopeptide domains [25]. The central triple-helix structural domains of both CSSC and NTSC α1-chain and α2-chain consisted of 339 uninterrupted Gly-X-Y triplets (1017 residues). As shown in Figures S1 and S2, the CSSC α1-chain consisted of 1449 amino acids, with a (Gly-X-Y)_339_ conserved sequence located at G^164^-G^1180^ accounting for 70.19% of the total amino acid content of the α1-chain, while the CSSC α2-chain consisted of 1352 amino acids, with a (Gly-X-Y)_339_ conserved sequence located at G^81^-G^1097^ accounting for 75.22% of the total amino acid content of the α2-chain. The NTSC α1-chain consisted of 1447 amino acids, with a (Gly-X-Y)_339_ conserved sequence located at G^163^-G^1179^ accounting for 70.28% of the total amino acid content of the α1-chain, while the NTSC α2-chain consisted of 1350 amino acids and the conserved sequence of (Gly-X-Y)_339_ was located at G^79^-G^1095^ and accounted for 75.33% of the total amino acid content of the α2-chain. These results indicated that CSSC and NTSC contained similar conserved amino acid sequences. In addition, the sequence comparison between the α1-chain and the α2-chain of CSSC and NTSC (Figures S3 and S4) revealed that the amino acid sequence differences were concentrated in the central triple-helical domain of the α-chain. For the Gly-X-Y triplet repeat sequence, E and F, which occur frequently in the second position, and K and R in the third position, are usually conserved, while variations occur generally between amino acids with hydrophobic side chains (A, P, and V) and amino acids with polar uncharged side chains (S, T, G, and N) in the second and third positions [26]. Differentiation of amino acids in conserved Gly-X-Y sequences in fish from different living environments might have implications for their physicochemical and functional properties.

The amino acid composition of collagen varies from species to species and is influenced by environmental factors, particularly temperature [27]. Analysis of the amino acid sequences of CSSC and NTSC showed the highest content of glycine, which is generally present uniformly every three residues of most collagen molecules [2], accounting for approximately one-third of the total amino acids (28.1% for CSSC α1, 30.0% for CSSC α2, 26.7% for NTSC α1, and 28.5% for NTSC α2). Notably, since hydroxyproline is formed by hydroxylation of proline and has the same primary structure as proline [28], the P content of collagen amino acid sequences in all current databases corresponds to the sum of proline and hydroxyproline in collagen [25]. Imino acids (proline + hydroxyproline) help to strengthen the triple-helix structure of collagen by forming hydrogen bonds and increasing the thermal stability of collagen [18]. The CSSC α1-chain imino acid content was 14.8%, and that of the α2-chain was 13.8%, while the corresponding content of the NTSC α1-chain was 17.4%, and that of the α2-chain was 16.9%. The imino acid content of NTSC was significantly higher than that of CSSC, suggesting that NTSC was more stable than CSSC. These experimental results also suggested that habitat temperatures were a critical factor in determining the imino acid content of fish collagen, with higher habitat temperatures being associated with higher imino acid content [2]. In addition, the total hydrophobic amino acid (W, A, V, L, I, P, F, M) [29] content of NTSC (39.95% for α1-chain and 38.82% for α2-chain) was also higher than that of CSSC (36.43% for α1-chain and 35.07% for α2-chain), indicating that the emulsifying and foaming properties of NTSC would be more favorable than those of CSSC [3]. The difference in amino acid content would affect the functional properties of collagen and was an important characteristic that would determine the potential applications of collagen.

#### 3.2.3. UV

**Figure 2:**
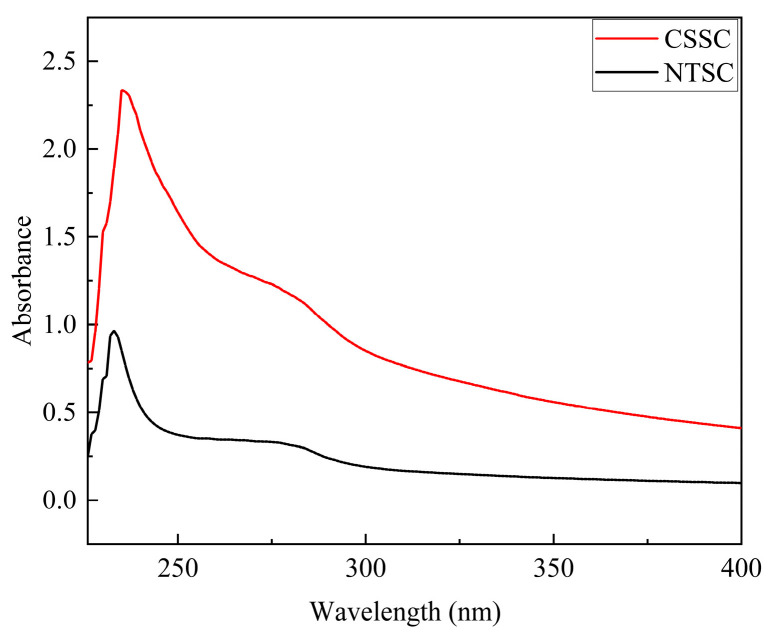
UV spectra of CSSC and NTSC.

The UV absorption spectra of CSSC and NTSC in the wavelength range of 190–400 nm are shown in Figure 2. Due to the presence of chromogenic groups such as C=O, -COOH, and CO-NH_2_ in the triple-helix polypeptide chain of collagen, the maximum UV absorption peak was observed near 230 nm. Due to the small amount of aromatic amino acids such as phenylalanine and tyrosine in the collagen, the absorption peak near 280 nm was relatively weak [30]. CSSC had a maximum absorption peak at 235 nm; NTSC had a maximum absorption peak at 233 nm. This pattern was similar to the results reported in previous studies for black carp skin collagen [5], yellowfin skin collagen [23], and Pacific cod skin collagen [31].

#### 3.2.4. FTIR

**Figure 3:**
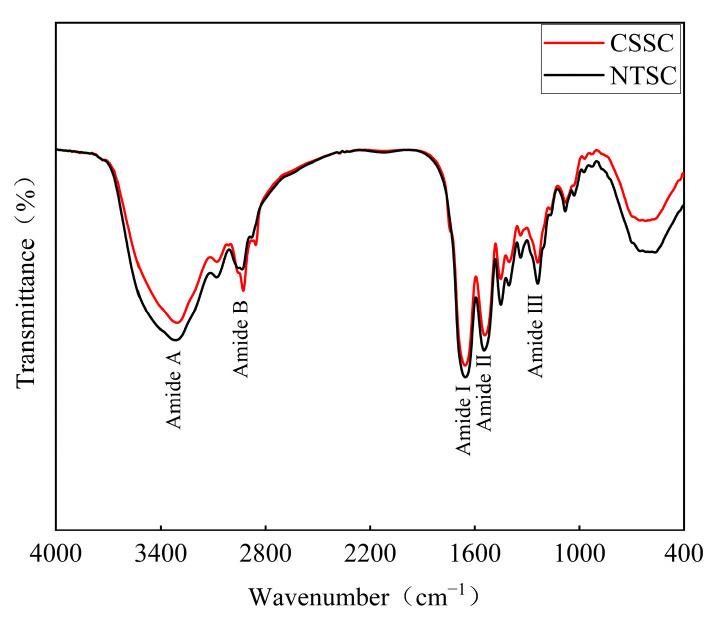
FTIR spectra of CSSC and NTSC.

As shown in Figure 3, both CSSC and NTSC contained five major characteristic absorption peaks. The amide A band was observed at 3306.99 cm^−1^ and 3315.51 cm^−1^; the amide B band was observed at 2927.83 cm^−1^ and 2934.90 cm^−1^; the amide I band was observed at 1655.73 cm^−1^ and 1654.16 cm^−1^; the amide II band was found at 1542.55 cm^−1^ and 1546.34 cm^−1^, and the amide III band was found at 1238.87 cm^−1^ and 1238.91 cm^−1^ for CSSC and NTSC, respectively. The slight differences in the positions and intensities of the infrared absorption peaks were attributed to differences in the secondary structure of collagen [24]. The FTIR spectra of CSSC and NTSC were similar to the results for type I fish skin collagen extracted from sturgeon fish [2], black ruff [30], and black carp [5], indicating that both CSSC and NTSC were type I collagen. In addition, the absorbance ratio of the amide III band to the wave number at 1450 cm^−1^ was close to 1 for CSSC and NTSC, indicating that the fish skin collagen prepared in this study maintained its intact triple-helix structure [2].

#### 3.2.5. FTIR Spectral Curve Fitting

To analyze the structural differences between CSSC and NTSC, we determined the secondary structure parameters of collagen using spectral curve fitting. The amide I band region was from the stretching vibration of C=O in the peptide backbone, and the hydrogen bond formed between C=O and adjacent groups was barely affected by the side chain conformation, a structural pattern that could maintain the stability of the collagen triple helix; therefore, this spectral region was used for collagen secondary structure analysis, including α-helices (1650–1660 cm^−1^), β-sheets (1610–1642 cm^−1^ and 1680–1700 cm^−1^), β-turns (1660–1680 cm^−1^), and random coils (1642–1650 cm^−1^) [32]. The difference in the protein secondary structure was related to the local sequence of amino acids and intermolecular interactions [33].

**Figure 4:**
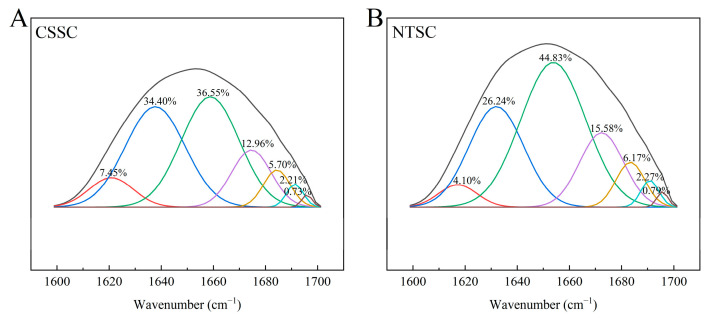
Curve-fitting analysis of amide I band: (**A**) CSSC and (**B**) NTSC. The black line is the FTIR spectra of collagen amide I band (1600–1700 cm^−1^).

As shown in Figure 4, the spectral curve fits of CSSC and NTSC collagen both included seven bands related to the secondary structure, with correlation coefficients of 0.99950 and 0.99945, respectively, indicating that these were good fits and could be used to analyze the secondary structure of collagen. The α-helix structure was tight and stable, an important factor in protein folding and biomaterial design [17]. The results showed that the α-helix content of CSSC (36.55%) was significantly lower than that of NTSC (44.86%), suggesting that NTSC was more stable, a pattern that may be related to the fact that chum salmon is a cold-water fish with a narrow temperature range, whereas tilapia is a warm-water freshwater fish that is adapted to changing environments. The β-sheet structure was less stable than the α-helix, and its content was negatively correlated with the surface hydrophobicity of the protein [33]. The higher β-sheet content of CSSC (50.49%) compared to NTSC (39.56%) could indicate that NTSC had a higher surface hydrophobicity corresponding to the high content of hydrophobic amino acids in the amino acid sequence of NTSC and would affect the functional properties of the collagen. β-turns and random coils are usually associated with protein defolding, dissociation, and rearrangement [18]. The β-turn content of CSSC (12.96%) was lower than that of NTSC (15.58%), and neither contained random coils. α-helices and β-sheets are generally considered to be “ordered” secondary structures, whereas β-turns and random coils are often considered to be “disordered” protein secondary structures [17]. The results showed that the secondary structure components of CSSC and NTSC were similar, with the ordered structure being significantly more prominent than the disordered structure, indicating that the secondary structures of both CSSC and NTSC were tightly connected.

#### 3.2.6. XRD

**Figure 5:**
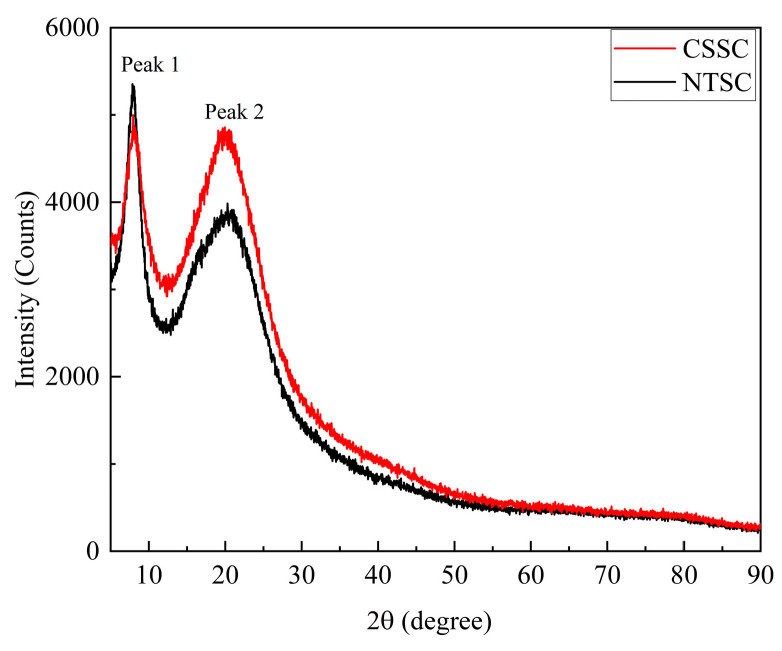
X-ray spectra of CSSC and NTSC.

As shown in Figure 5, both CSSC and NTSC had two collagen characteristic peaks. The diffraction angles corresponding to the collagen peaks were 7.94° and 19.94° for CSSC and 8.06° and 20.30° for NTSC, respectively. The first sharp peak was associated with the triple-helix structure of collagen and reflected the distance between the molecular chains of the collagen fibrils, while the second broad, rounded peak was the result of diffusion between the layers of the collagen fibrils and reflected the distance between the collagen skeleton [31]. The structure of collagen was analyzed using the Bragg equation; the distance between collagen molecular chains was 11.1 Å for CSSC and 11.0 Å for NTSC, and the distance between collagen skeletons was 4.45 Å for CSSC and 4.37 Å for NTSC. The molecular chain distance and collagen skeleton distance observed for CSSC were greater than those observed in NTSC, suggesting that CSSC might be a better vehicle for drug delivery than NTSC [16].

### 3.3. Td

**Figure 6:**
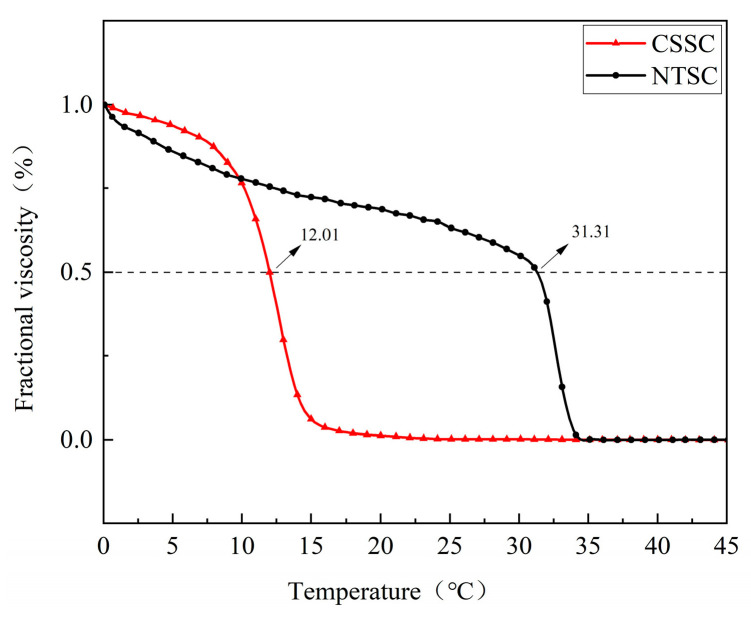
Thermal denaturation curves of CSSC and NTSC.

The thermal stability of collagen is described by the Td value in solution, and the temperature at which the viscosity is reduced to half of the original value is called the Td [4]. As shown in Figure 6, chum salmon were anadromous cold-water fish that usually live at 8–12 °C, and the skin collagen Td value was 12.01 °C, similar to the values of other cold-water fish such as Pacific cod (14.5 °C) [31], skipjack tuna (17.8 °C) [24], Spanish mackerel (15.12 °C) [4], and Hake (10 °C) [34]. Tilapia, a warm-water fish, had a collagen Td value of 31.31 °C, higher than those of silver carp (29 °C) [27] and sturgeon fish (29.34 °C) [2]. The NTSC Td values were much higher than those of CSSC, a result that was consistent with the report of Rose et al. [35] demonstrating that the thermal stability of warm-water fish collagen is higher than that of cold-water fish collagen. These experimental results suggest that the temperature of the fish living environment was a significant determinant of the Td of fish collagen [34]. NTSC had a higher Td, and it was more stable, suggesting a promising application in the development of biofunctional materials and pharmaceuticals. In contrast, CSSC had a low Td value, and its viscosity decreased rapidly at room temperature and thus was consumed rapidly after defrosting, and thus it could be used as a film and coating for the preservation of frozen and refrigerated foods [36].

### 3.4. Rheological Properties

The rheological properties of collagen solutions are important processing characteristics. Dynamic frequency sweep tests are primarily used to measure the viscoelastic behavior of materials as a function of frequency. Steady shear tests are used to measure viscosity as a function of shear rate to describe the flow properties of the material.

#### 3.4.1. Dynamic Frequency Sweep Tests

**Figure 7:**
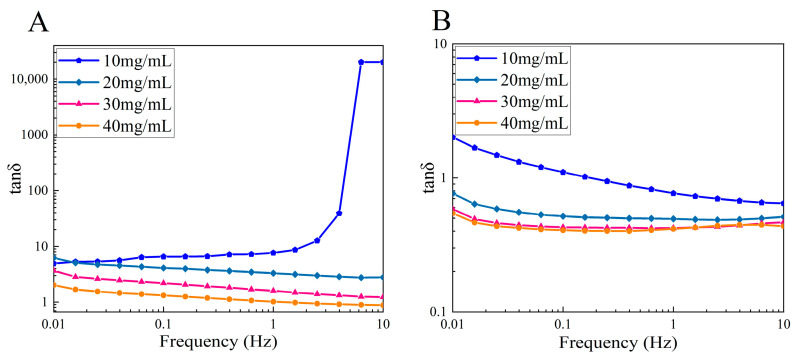
Effect of concentration on dynamic rheology of collagen solutions: (**A**) loss factor (tan *δ*) of CSSC and (**B**) loss factor (tan *δ*) of NTSC.

As shown in Figure 7, the loss factor (tan *δ* = loss modulus/storage modulus) of CSSC and NTSC with different concentrations showed typical viscoelastic behavior with frequency. A tan *δ* < 1 indicated a predominantly elastic behavior, while tan *δ* > 1 indicated a more viscous behavior [37]. The results showed that tan *δ* decreased with the increased concentration of CSSC and NTSC, indicating that the storage modulus rose more rapidly than the loss modulus, and the contribution of elasticity to the solution system increased. In addition, the value of tan *δ* decreased with increased frequency, indicating that the properties of the solution changed from largely viscous behavior to more elastic behavior with the increase in frequency [18]. Comparing CSSC and NTSC, NTSC tan *δ* values were lower at the same frequency and concentration, a result that was due to a higher degree of entanglement of the chain segments of NTSC collagen molecules and greater internal friction in the movement of the chain segments resulting in a blockage of the chain segment movement, so that NTSC exhibited greater elastic properties. The data showed that CSSC was dominated by viscous behavior, and the properties of the solution changed from viscous to elastic only when the concentration rose to 40 mg/mL and the frequency was higher than 1.00 Hz, while NTSC was dominated by elastic behavior, and the properties of the solution changed to viscous when the concentration was lower than 10 mg/mL and the frequency was lower than 0.16 Hz.

**Figure 8:**
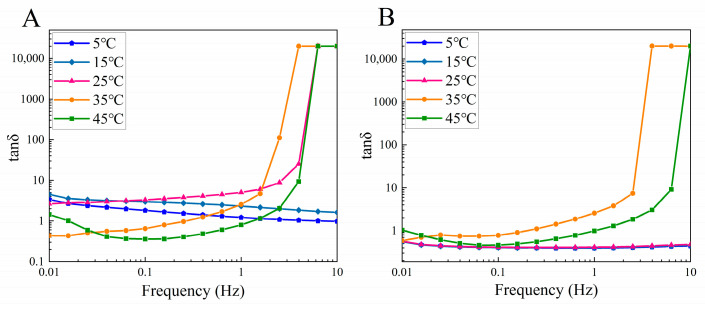
Effect of temperature on dynamic rheology of collagen solutions: (**A**) loss factor (tan *δ*) of CSSC and (**B**) loss factor (tan *δ*) of NTSC.

The effect of temperature on the rheological properties of the collagen solutions is shown in Figure 8. For CSSC, at temperatures below 15 °C, the collagen solution tan *δ* increased with increasing temperature, possibly caused by the denaturation of the collagen triple-helical structure, where the increase in temperature intensified the thermal movement of the collagen molecules, disrupting the hydrogen bonds that stabilize the collagen structure [38]. For NTSC, the tan *δ* of the collagen solution did not change much at temperatures below 25 °C, indicating that the collagen solution was stable in its viscous and elastic properties below the Td. At temperatures below the Td, CSSC and NTSC showed different rheological behavior with increasing temperature, probably due to CSSC and NTSC having different inter- and intramolecular hydrogen bonds and large differences in their amino acid composition [22]. When the temperature was further increased above Td, the tan *δ* of CSSC and NTSC solutions lost regularity. At this point, the collagen had been denatured, and the non-covalent bonds that keep the triple-helix structure stable were broken, with the result that the collagen molecular structure became random and irregular [22]. Overall, below the Td, the structure of the CSSC solution changed with temperature, while the NTSC structure remained stable.

#### 3.4.2. Steady Shear Tests

**Figure 9:**
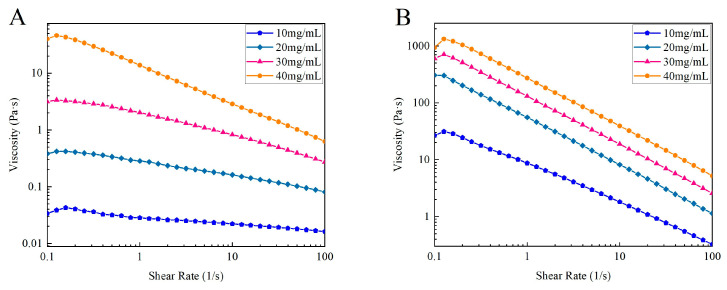
Effect of concentration on steady state rheology of collagen: (**A**) viscosity and shear rate curves of CSSC at different concentrations and (**B**) viscosity and shear rate curves of NTSC at different concentrations.

Figure 9 shows the viscosity versus shear rate for different concentrations of CSSC and NTSC solutions at 10 °C. The viscosity of CSSC and NTSC solutions decreased with increasing shear rate, and all collagen solutions of different concentrations exhibited typical shear thinning behavior of non-Newtonian fluids, consistent with the results of previous studies [19]. In addition, the viscosity of CSSC and NTSC solutions increased with increasing concentration due to the increased entanglement between collagen molecule chains and greater intermolecular friction [17]. The rheological parameters of the collagen solutions correlate with the Ostwald model (R^2^ > 0.9), and the results are shown in Table 1. The *n* values were less than 1, indicating a strong pseudoplasticity of both collagen solutions [19]. *K* was directly related to viscosity and could be used to indicate the viscosity properties of a fluid under certain conditions [39]. The *K* values of CSSC and NTSC increased with the concentration, and for the same concentration of collagen solution, the *K* values of NTSC were much higher than those of CSSC, indicating that NTSC had a higher viscosity. The experimental results were consistent with the report of Rose et al. [35], who found that the viscosity of warm-water fish collagen was higher than that of cold-water fish collagen. Viscosity was related to molecular conformation and relative molecular weight [5]; due to the difference in living environments, CSSC had a lower molecular weight and lower crosslinkage, factors that may contribute to the low viscosity of CSSC.

**Figure 10:**
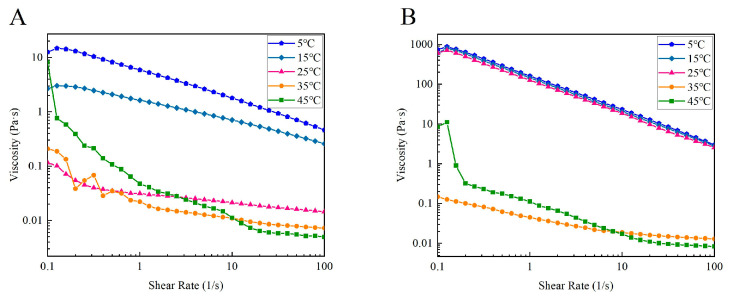
Effect of temperature on steady state rheology of collagen: (**A**) viscosity and shear rate curves of CSSC at different temperatures and (**B**) viscosity and shear rate curves of NTSC at different temperatures.

Figure 10 shows the viscosity versus shear rate of CSSC and NTSC solutions at different temperatures at concentrations of 30 mg/mL. Below 15 °C, the viscosity of CSSC solution decreased with increasing temperature, and below 25 °C, the viscosity of NTSC solution decreased only slightly with increasing temperature. The results reflected the irregular thermal motion of the collagen molecules increasing with increasing temperature, resulting in a decrease in the viscosity of the solution. At the same time, the apparent viscosity decreased with increasing shear rate, indicating that all samples exhibited shear thinning behavior of non-Newtonian fluids [19]. However, when the temperature was above the Td, the viscosity of CSSC and NTSC solutions changed irregularly as the shear rate increased and the temperature increased, and the triple helix of the collagen began to unravel, and thus denaturation occurred. Below the Td, as shown in Table 2, the Ostwald model fits showed *n* values less than 1, indicating that both collagen solutions had strong pseudoplasticity, with the *K* values of CSSC decreasing more with increasing temperature than NTSC. Thus, the viscosity of CSSC changed more dramatically with temperature.

Differences in habitat water temperatures could lead to different rheological behavior of fish skin collagen solutions, a factor that could lead to applications in different fields. At low temperatures, the CSSC solution had low viscosity and behaved as a viscous fluid, and thus would be suitable for beverage development applications, while the NTSC solution had high viscosity and displayed elastic behavior, and thus could be used as a food additive for solid food processing applied to meat and bakery products.

### 3.5. Functional Properties

#### 3.5.1. WHC

The WHC values of CSSC and NTSC were 3.57 ± 0.23 g/g and 34.13 ± 0.10 g/g, respectively, higher than those of sour cherry kernel protein concentrate (2.42 ± 0.09 g/g) [21], casein (2.48 ± 0.11 g/g) [20], and chicken feet collagen (1.9 ± 0.1 g/g) [40]. The poor WHC of CSSC, indicating that it dissolved more readily in water, corresponded to the finding that highly soluble proteins had a lower water absorption capacity [41], whereas NTSC had a high WHC, possibly related to the hydrophilic amino acid content and the size and shape of the protein [42]. A high WHC facilitated cell growth, and thus NTSC could be used as a wound dressing or cosmetic material in pharmaceutical and cosmetic applications [43].

#### 3.5.2. OHC

The OHC values of CSSC and NTSC were 15.6 ± 0.13 g/g and 28.23 ± 0.28 g/g, respectively, higher than those of sour cherry kernel protein concentrate (1.73 ± 0.17 g/g) [21], soft-shelled turtle collagen [43], and chicken feet collagen (5.3 ± 0.3 g/g) [40]. NTSC had a greater OHC, possibly related to the presence of more non-covalent bonds in NTSC such as hydrophobic force, electrostatic force, and hydrogen bonds involved in lipoprotein interactions [42]. Proteins with higher OHC hold their shape well in food, and thus both CSSC and NTSC could be used in the meat or confectionery industries [43].

#### 3.5.3. FC and FS

FC indicates the ability of a protein solution to froth, and FS measures the ability of the foam to remain intact and undamaged. The FC of CSSC was 20.40 ± 0.40%, and the FS was 41.16 ± 1.15%, while the FC of NTSC was 23.87 ± 0.23%, and the FS was 96.65 ± 0.03%. The FC of NTSC was greater than that of CSSC, a result that might be related to the large molecular weight of NTSC, making it easier for the collagen to enter the air–liquid interface and thus increase FC [44]. FS was determined by the physical properties of the film formed, and the greater FS of NTSC might be related to its higher molecular weight that would allow it to form more stable protein films [42]. In addition, FS might be related to the fact that NTSC solutions were more viscous, and thus more conducive to the formation of multi-layer protein filmed at the interface [3]. Both CSSC and NTSC had higher FC values than casein (3.95–10.15%) [20] and black ruff skin collagen (15 ± 1.26%) [30], and greater FS than soft-shelled turtle collagen [43], casein (0.17–0.54%) [20], and black ruff skin collagen (10 ± 2.1%) [30]. The high FC and FS of CSSC and NTSC indicated that they could be used in the food processing industry to assist in the formation and stabilization of foam structures, while the FS of NTSC might be more suitable for use in solid food processing industries such as confectionery and puffed foods where the need for stability is greater, while CSSC would be more suitable for use in the production of sparkling beverages such as beer and wine that have a short-term aerated state [45].

#### 3.5.4. EAI and ESI

Collagen has surface activity, and thus is widely used as food emulsifiers and stabilizers due to its emulsifying properties; collagen thus plays an important role in food development [30]. Collagen reduced the surface tension by adsorption at the interface, forming a thick adsorbed layer with good interfacial rheology, delaying the breakup of the film and improving the stability of the emulsion [44]. The EAI of CSSC was calculated as 85.18 ± 0.23 m^2^/g, with an ESI of 34.86 ± 0.43 min, while the EAI of NTSC was 86.56 ± 0.13 m^2^/g, with an ESI of 84.63 ± 2.22 min. The EAI of NTSC was slightly greater than that of CSSC, while the ESI was much higher than that of CSSC, results that were associated with surface hydrophobicity, where the high surface hydrophobicity allowed faster protein adsorption to the oil–water interface and stronger protein–protein interactions at the interface for better EAI [33]. In addition, the greater viscosity and higher molecular weight of NTSC could also improve the ESI [3]. The EAI values of both CSSC and NTSC were higher than those of black ruff skin collagen (19.04 ± 0.02 m^2^/g) [30], sour cherry kernel protein concentrate (38.91 ± 2.50 m^2^/g) [21], and yellowfin skin collagen (37.0 ± 0.33 m^2^/g) [23], and the ESI was higher than that of black ruff skin collagen (14.37 ± 0.14 min) [30], sour cherry kernel protein concentrate (37.49 ± 2.41 min), and peanut protein concentrate (19.18 min) [21]. The results indicated that both types of extracted fish skin collagen could be used as emulsifiers in food development, while NTSC could also be used as an emulsion stabilizer in the food industry.

## 4. Conclusions

In this study, CSSC was successfully extracted from chum salmon skin and NTSC was successfully extracted from Nile tilapia skin. Both collagens were further identified by SDS-PAGE, UV, FTIR, and XRD as type I collagen with an intact triple-helix structure. As shown by the comparison of collagen structures, CSSC and NTSC contained similar (Gly-X-Y)_339_ conserved sequences. However, the molecular weight, β- and γ-chain content, imino acid content, and α-helices and β-turns contents of CSSC were lower than those of NTSC, suggesting that NTSC had stronger intra- and intermolecular crosslinks and that its stability was greater. The difference in collagen structure would affect thermal stability as well as rheological and functional properties. The Td value of CSSC was 12.01 °C, and that of NTSC was 31.31 °C, indicating that the thermal stability of the collagen from the warm-water species was higher than that of the collagen from the cold-water species. The results of dynamic frequency sweep tests revealed that CSSC displayed viscous behavior, and the collagen structure varied with temperature, whereas NTSC displayed a more elastic behavior, and the collagen structure remained stable with temperature. The results of steady shear tests revealed that the viscosity of CSSC was lower than that of NTSC, and that the viscosity of CSSC varied more dramatically with temperature. The results of the functional experiments showed that both CSSC and NTSC had high values of OHC, FC, and EAI. Moreover, NTSC had greater WHC, FS, and ESI values than CSSC. Overall, both CSSC and NTSC displayed potential applications in food processing and biomedicine, with CSSC being more appropriate for applications in the preservation of frozen and refrigerated food materials and sparkling beverage production, while NTSC would have greater potential for applications in biofunctional materials and solid food processing.

Differences in fish habitat water temperatures affected the structure of collagen, which in turn affected their physicochemical and functional properties, and consequently their direction of application. Investigating the differences in physicochemical and structural characteristics of skin collagen between cold-water and warm-water fish could provide reference data for the processing and industrial application directions of fish skin collagen from different habitat water temperature and would help in selecting suitable fish sources for collagen extraction, thus achieving high-value utilization of fish byproducts.

## Figures and Tables

**Table 1:** Rheological parameters from the Ostwald model with different concentrations.

Sample	Concentration (mg/mL)	*K*	*n*	R^2^
CSSC	10	0.030	0.870	0.942
	20	0.273	0.777	0.974
	30	1.864	0.693	0.968
	40	14.416	0.459	0.969
NTSC	10	8.941	0.435	0.975
	20	57.900	0.242	0.994
	30	148.581	0.301	0.970
	40	319.440	0.376	0.933

**Table 2:** Rheological parameters from the Ostwald model with different temperatures.

Sample	Temperature (°C)	*K*	*n*	R^2^
CSSC	5	5.863	0.567	0.970
	15	1.585	0.680	0.972
	25	0.036	0.613	0.810
	35	0.013	−0.224	0.919
	45	0.000	−8.575	0.991
NTSC	5	184.941	0.303	0.969
	15	168.724	0.318	0.951
	25	141.830	0.279	0.972
	35	0.048	0.533	0.989
	45	0.038	−1.427	0.776

## Data Availability

The original contributions presented in the study are included in the article and Supplementary Materials, further inquiries can be directed to the corresponding authors.

## Supplementary Materials

The following supporting information can be downloaded at the following: https://www.mdpi.com/article/10.3390/foods13081213/s1, Figure S1: Primary structure identification of type I collagen. (A) *Oncorhynchus keta* type I collagen α1-chain; (B) *Oncorhynchus keta* type I collagen α2-chain; Figure S2: Primary structure identification of type I collagen. (A) *Oreochromis niloticus* type I collagen α1-chain; (B) *Oreochromis niloticus* type I collagen α2-chain; Figure S3: Sequence alignment of type I collagen α1-chain between CSSC and NTSC; Figure S4: Sequence alignment of type I collagen α2-chain between CSSC and NTSC; Table S1: LC-MS/MS identification of α1 and α2 chains.
