# Supplementary material for: Comparison of Structural and Physicochemical Characteristics of Skin Collagen from Chum Salmon (Cold-Water Fish) and Nile Tilapia (Warm-Water Fish)
Source: Foods. 2024 Apr 16;13(8):1213. doi: 10.3390/foods13081213 (PMC11049058; doi:10.3390/foods13081213)
Supplement: Supplementary file 1 [file foods-13-01213-s001.zip › foods-2964508-supplementary.pdf]

Supplementary Materials

Comparison of Structural and Physicochemical Characteristics of Skin Collagen from Chum Salmon (Cold-Water Fish) and Nile Tilapia (Warm-Water Fish)

Yan Zheng [1], Yushuang Li [1], Cong Ke [1], Xiyuan Gao [1], Zhiyu Liu [2,*] and Junde Chen [1,*]

1 Technical Innovation Center for Utilization of Marine Biological Resources, Third Institute of Oceanography, Ministry of Natural Resources, Xiamen 361005, China; zhengyan@tio.org.cn (Y.Z.); liyushuang@tio.org.cn (Y.L.); 13172508013@163.com (C.K.); gxygxy18649687417@163.com (X.G.)

2 Fisheries Research Institute of Fujian, Key Laboratory of Cultivation and High-Value Utilization of Marine Organisms in Fujian, Xiamen 361021, China

* Correspondence: liuzy@fafu.edu.cn (Z.L.); jdchen@tio.org.cn (J.C.); Tel.: +86-0592-2195527 (J.C.)

```
1    MFSFVDIRLA LLLSATVLLA RGQGEDDRTA GSCTLDGQYY NDRDVWKPEP
51   CQICVCDSGT VMCDEVICED TSDCPNPVIP HDECCPICPD DGFQEPKVEG
101  PQGDRGAKGE PGPAGFPGND GIPGQPGLPG PPGPPGPPGL GGNFSPQMSG
151  GFDEKSGGGM SMPGPMGPMG PRGPPGPPGS SGPQGFTGPP GEPGEAGSSG
201  PMGPRGPAGP PGKNGDDGES GKPGRPGERG ASGPQGARGF PGTPGLPGIK
251  GHRGFSGLDG AKGESGPAGP KGEGGASGEN GAAGAMGPRG LPGERGRAGP
301  NGAAGARGND GAAGAAGPPG PTGPAGAPGF PGGPGAKGEV GAQGARGGEG
351  PQGSRGEAGN PGPAGPAGLA GNNGADGNPG TKGAPGSAGI AGAPGFPGPR
401  GPPGPQGAGG APGPKGNTGE VGANGAKGEA GAKGESGPAG VQGPAGPAGE
451  EGKRGSRGEP GAAGARGAPG ERGGPGSRGF PGSDGAAGPK GGPGERGGAG
501  VAGAKGNTGE PGRNGEPGMP GSKGMTGSPG SPGPDGKTGP SGAGGQDGRP
551  GPPGPVGARG QPGVMGFPGP KGAAGEGGKP GERGVMGPGG AVGAPGKDGD
601  VGAPGAPGVA GPSGERGEQG AGGPPGFQGL SGPQGAIGET GKPGEQGLPG
651  EGGAPGSAGS RGDRGFPGER GAPGPSGPSG ARGSPGSAGN DGAKGEAGAA
701  GAPGGQGPPG LQGMPGERGA GGLPGLKGDR GDQGVKGADG AGGKDGVRGM
751  TGPIGPNGPA GSPGDKGETG APGAVGPSGA RGAPGERGES GAPGPAGFAG
801  PPGGDGQPGA KGEAGDNGAK GDGGAQGPAG PTGAPGPQGP AGNTGAKGAR
851  GAAGPPGATG FPGAAGRVGP PGPSGNSGPP GPPGPGGKEG QKGNRGETGP
901  AGRPGELGAA GPPGAQGEKG QPGGDGPNGP SGTPGPQGIG GQRGIVGLPG
951  QRGERGFPGL AGQLGEPGKQ GPGGPSGERG PPGPMGPPGL AGAPGEPGRE
1001 GTPGNEGSSG RDGAAGPKGE RGESGVAGAS GAPGPPGAPG SVGPAGKSGD
1051 RGESGPAGPA GIAGPAGPRG PSGPAGARGD KGEAGEAGER GMKGHRGFTG
1101 MQGPPGPSGQ SGESGPAGAS GPAGPRGPSG SAGAAGKDGM SGLPGPIGPP
1151 GPRGRSGEMG PSGPPGPPGP PGPPGPPGGG FDMGFIAQPX QEKAPDPFRH
1201 FRADDANVMR DRDLEVDTTL KSLSQQIENI RSPEGTKKNP ARTCRDLKMC
1251 HPDWKSGEYW IDPDQGCTQD AIKVYCNMET GETCVYPTEA XIPKKSWYTS
1301 KNIKEKKHVW FGEAMTDGFQ FEYGSEGSSA QDVNIQLTFL RLMATEASQN
1351 ITYHCKNSIA YMDQQSGNLK KSLLLQGSNE IEIRAEGNSR FTYSVTEDGC
1401 TSHTGAWGKT VIDYKTTKTS RLPIIDIAPM DVGAPNQEFG IEVGPVCFL
```

A

```
1    MLSFVDNRIL LLLAVTSFLA SCQSGPTGEK GPRGDRGPQG RNGRDGKDGL
51   PGVAGPPGPP GLGGNFAAQF DGGKGSDPGP GPMGLMGSRG PNGPPGSPGP
101  QGFTGHAGEP GEPGQTGSIG ARGPSGSAGK PGEDGNNGRP GKPGDRGGPG
151  TQGARGFPGT PGLPGMKGHR GYNGLDGRKG ESGSGGAKGE TGAHGANGNP
201  GPAGSRGLNG ERGRAGPAGP AGARGADGST GPAGPAGPLG AAGPPGFPGA
251  PGPKGEIGGA GANGPSGPQG GRGEPGTNGA VGPVGPVGNP GNNGINGAKG
301  AAGLPGVAGA PGFPGPRGGP GPQGPQGSTG ARGLGGDPGP SGQKGDSGAK
351  GEPGHSGVQG AAGPAGEEGK RGSTGEVGAT GPAGLRGARG GAGTRGLPGL
401  EGRGGPIGMP GARGATGPAG IRGAPGDAGR AGESGLTGAR GLPGNSGQGG
451  PPGKEGPSGA AGLDGRTGPP GPTGPRGQPG NIGFPGPKGP GGEAGKGGDK
501  GPTGATGLRG GPGADGNNGA PGPAGVVGNA GEKGEQGPSG APGFQGLPGP
551  AGPAGEAGKP GNQGMHGDQG LPGPAGVKGE RGNSGPAGSA GSQGAIGARG
601  PAGTPGPDGG KGEPGSVGIA GGPGHQGPGG MPGERGAGGT PGPKGEKGEG
651  GHRGLEGNMG RDGARGAAGP SGPPGPSGAN GEKGESGSFG PAGPAGLRGP
701  SGERGEGGPA GPPGFAGPPG SDGQSGPRGE KGPAGGKGDV GPAGPSGPAG
751  QSGPSGASGP AGPPGGRGDA GPSGLTGFPG AAGRVGGPGP AGISGPPGSA
801  GPAGKDGPRG LRGDSGPAGP QGEHGQIGPS GIAGDKGPTG ESGPPGAPGT
851  AGPQGVLGPS GFVGLPGSRG DKGLPGGPGA VGEPGRLGPA GASGPRGPAG
901  NIGMPGMTGT QGEAGREGSP GNDGPPGRPG TAGFKGDRGE PGSPGALGSS
951  GQPGPNGPSG AVGRPGNRGE SGPTGNGGPV GAAGARGAPG PAGPRGEKGG
1001 AGEKGDRGMK GLRGHGGLQG MPGPNGPSGE TGSAGITGPA GPRGPAGPHG
1051 PPGKDGRAGG HGAIGPVGHR GSPGHLGPAG PPGSPGLPGP AGPAGGGYDQ
1101 SGGYDEYRAD QPSLRAKDYE VDATIKSLNS QIENLLTPEG SKKNPARTCR
1151 DIKLSHPEWS SGFYWIDPNQ GCIADAIKAH CDFSTGHTCI HPHPESIARK
1201 NWYRSSENKK HVWFGETING GTEFAYNDET LSPQSMATQL AFMRLLANQA
1251 TQNITYHCKN SVAYMDGENG NLKKAVLLQG SNDVELSAEG NSRFTFNVLE
1301 DGCTRHTGQW SKTVIEYRTN KPSRLPILDI APLDIGGADQ EFGLDIGPVC
1351 FK
```

B

Figure S1. Primary structure identification of type I collagen. (A) *Oncorhynchus keta* type I collagen α1-chain; (B) *Oncorhynchus keta* type I collagen α2-chain. The matching peptide is marked in red and bold, and the central triple helix domain Gly-X-Y triplet is marked in gray shading.

```
1 MFSFVDLRLA LLLSAAVLLV RAQGEDDRTG KSCTLDGQVF ADRDVWKPEP
51 CQICVCDSGT VMCDEVICED TTDCPNPIIP HDECCPICPD DGFQEPQTEG
101 TVGARGPKGD RGLPGPPGRD GMPGQPGLPG PPGPPGPPGL GGNFSPQMSG
151 GYDEKSPAMP VPGPMGPMGP RGPPGPPGSS GPQGFTGPPG EAGEPGSPGP
201 MGPRGPAGPP GKNGEDGESG KPGRPGERGP PGPQGARGFP GTPGLPGIKG
251 HRGFSGLDGA KGDTGPAGPK GEAGTPGENG TPGAMGPRGL PGERGRAGAT
301 GAAGARGNDG AAGAAGPPGP TGPAGPPGFP GGPGAKGDAG AQGGRGPEGP
351 AGARGEPGNP GPAGPAGPAG NPGSDGAPGA KGAPGAAGVA GAPGFPGPRG
401 PSGPQGAAGA PGPKGNTGEA GAPGSKGEAG AKGEAGAPGV QGPPGPPGEE
451 GKRGARGEPG AAGARGGPGE RGAPGGRGFP GSDGPAGPKG ATGERGAPGL
501 VGPKGATGEP GRTGEPGLPG AKGMTGSPGN PGPDGKIGPS GAPGQDGRPG
551 PPGPGGARGQ PGVMGFPGPK GAAGEAGKPG ERGTMGPTGP AGAPGKDGDV
601 GAQGPPGPAG PAGERGEQGP AGSPGFQGLP GPQGAVGETG KPGEQGVPGE
651 AGAPGPAGAR GDRGFPGERG APGAIGPAGA RGSPGASGND GAKGDAGAPG
701 TPGAQGPPGL QGMPGERGAA GLPGLRGNRG DQGPKGADGT PGKDGPRGLT
751 GPIGLPGPAG SPGDKGEPGA QGPVGPSGAR GPPGERGEAG PPGPAGFAGP
801 PGADGQPGAK GEPGDNGAKG DSGPPGPAGP TGAPGPQGPV GNTGPKGARG
851 PAGPPGATGF PGAAGRVGPP GPAGNAGPPG PPGPAGKEGP KGNRGETGPA
901 GRPGELGAAG PPGPPGEKGS PGADGAPGSA GIPGPQGIAG QRGIVGLPGQ
951 RGERGFPGLA GPVGEPGKQG PSGPSGERGP PGPMGPPGLA GAPGEPGREG
1001 TPGNEGAAGR DGAPGPKGDR GESGPAGAPG APGPPGAPGP VGPAGKTGDR
1051 GETGPAGPAG AAGPAGPRGP AGAPGLRGDK GETGEAGERG MKGHRGFTGM
1101 QGPPGPPGTS GESGPAGAAG PAGPRGPSGA AGAPGKDGVS GLPGPTGPPG
1151 PRGRSGEMGP AGPPGPPGPP GAPGAPGGGF DLGFMVQPQE KAPDPFRMYR
1201 ADDANVLRDR DLEVDSTLKS LSQQIEQIRS PDGTRKNPAR TCRDLKMCHP
1251 DWKSGEYWID PDQGCTQDAI KVYCNMETGE TCVSPTQREV AKKNWYISKN
1301 IKEKKHVWFG EAMNEGFQFE YGSEGSLPED VNIQMTFLRL MSTEASQNIT
1351 YHCKNSVAYM DAAAGNLKKA LLLQGSNEIE IRAEGNSRFT YSVLEDGCTS
1401 HTGTWGKTVI DYKTSKTSRL PIIDIAPMDV GAPDQEFGFE VGPVCFL
```

A

```
1 MLSFVDTRIL LLLAVTSYLA SCQFAGPKGP RGDRGPPGPN GKDGLPGPPG
51 PAGPPGPPGL GGNFAAQYDG VKAPDPGPGP MGLMGPRGPP GPPGASGPQG
101 HTGHAGEPGE PGQAGAVGPR GPPGPPGKAG EDGNNGRPGK PGDRGAPGPQ
151 GARGFPGTPG LPGMKGHRGY TGLDGRKGEP GAAGPKGEPG AHGAAGSPGL
201 AGARGLPGER GRPGPAGPAG ARGADGNAGP TGPAGPLGAA GPPGFPGGPG
251 PKGETGPVGA TGPSGPQGSR GEPGPNGAVG PVGPSGNPGA NGLNGAKGAA
301 GTPGVAGAPG FPGPRGGPGP QGPQGAAGPR GLAGDPGIQG VKGDSGPKGE
351 PGHSGPQGPP GPQGEEGKRG PTGEIGATGL AGARGARGAP GSRGMPGAEG
401 RTGPVGMPGA RGATGAAGPR GPPGDAGRAG EPGAAGLRGL PGSPGSSGPP
451 GKEGPAGPSG QDGRSGPPGP SGPRGLSGNI GFPGPKGPSG EPGKPGERGA
501 TGPTGLRGPP GPDGNNGATG ATGVAGGPGE KGEQGPSGSP GFQGLPGPAG
551 PTGEAGKPGD RGIPGEPGAA GNAGAKGERG NPGAAGSAGP QGPIGPRGPA
601 GAPGPDGGKG EPGPAGVAGA PGHQGAGGMP GERGGAGTPG PKGEKGEPGH
651 KGPDGNPGRD GPRGLAGPAG PPGPTGANGD KGEGGSFGPA GPAGPRGPSG
701 ERGEVGPAGA PGFAGPPGAD GQAGARGERG PSGAKGEVGP SGLAGPAGQS
751 GPAGPAGPGG PPGARGDNGP PGLTGFPGAA GRVGAAGPAG IVGPPGPAGP
801 SGKDGPRGPR GDPGPSGPSG EPGIIGPPGL AGEKGPSGES GPPGSPGAPG
851 TSGPLGLQGF VGLPGSRGDR GAPGGAGGVG EPGRLGPAGP PGARGAPGNI
901 GLPGMTGPQG EAGREGSPGN DGPPGRPGAA GLKGDRGEPG SAGTTGLAGA
951 PGPAGPTGAA GRPGNRGEAG PSGPSGAVGP AGARGASGPA GPRGEKGVAG
1001 DKGERGMKGL RGHPGLQGMP GPSGPPGDTG AAGAHGPSGP RGPAGPHGPV
1051 GKDGRPGAHG TMGAPGARGP NGYSGPVGPP GPPGLPGPPG PAGGGYDVSG
1101 GYDEYRADQP ALRAKDYEVD ATIKSLNTQI ENLLTPEGSR KNPARTCRDI
1151 KLSHPDWSSG FYWIDPNQGC TNDAIKVFCD FTTRETCIYA HPESIARKNW
1201 YRSTENKKHV WFGETINGGT EFTYNDETLS PQSMATQLAF MRLLSNQASQ
1251 NITYHCKNSV AYMDGESGSL KKAVVLQGSN DVELRAEGNS RFTFSVLEDG
1301 CTTHTGEWSK TVIEYRTNKP SRLPILDIAP LDIGGADQEF GLDIGPVCFK
```

B

Figure S2. Primary structure identification of type I collagen. (A) *Oreochromis niloticus* type I collagen α1-chain; (B) *Oreochromis niloticus* type I collagen α2-chain. The matching peptide is marked in red and bold, and the central triple helix domain Gly-X-Y triplet is marked in gray shading.

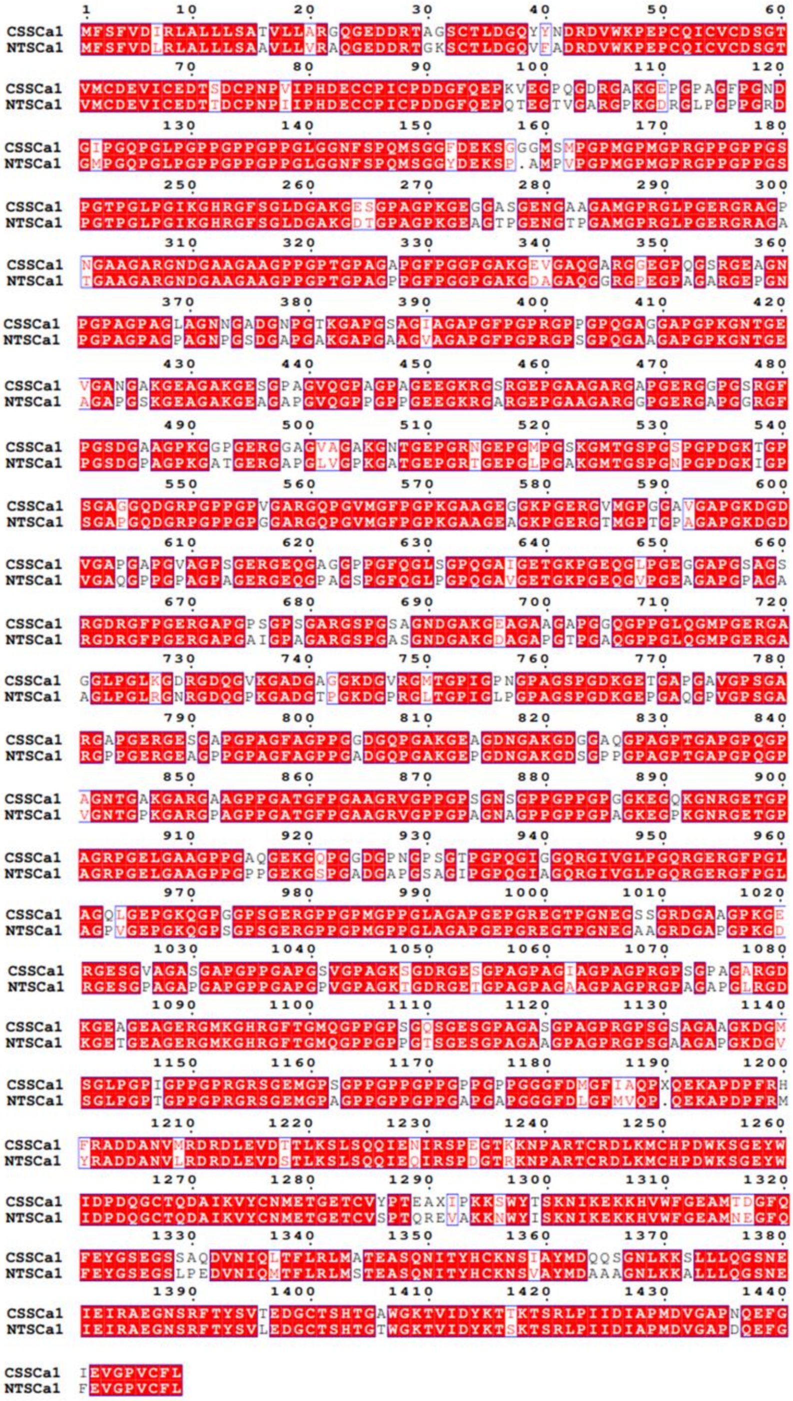

Figure S3. Sequence alignment of type I collagen α1-chain between CSSC and NTSC. The same amino acid is marked with red shading.

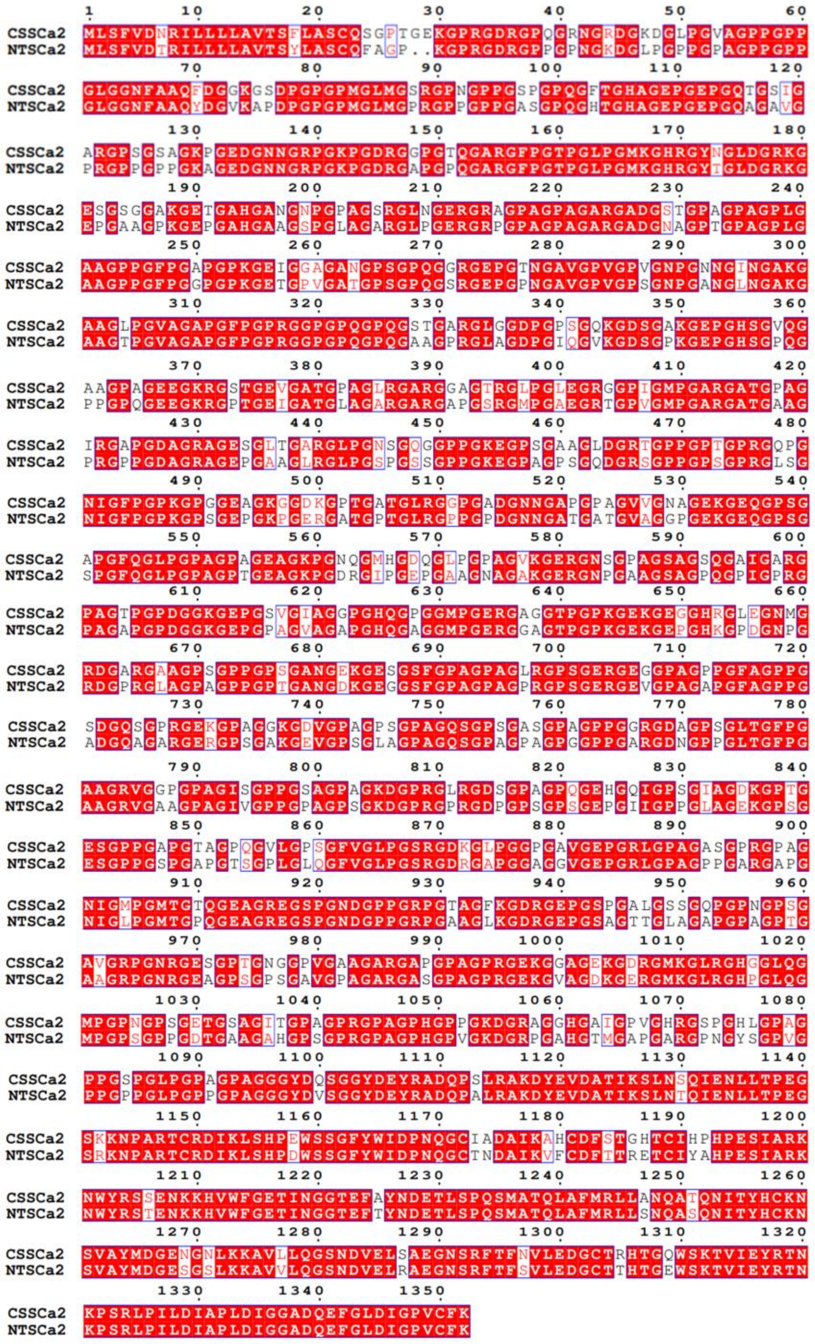

Figure S4. Sequence alignment of type I collagen α2-chain between CSSC and NTSC. The same amino acid is marked with red shading.

Table S1. LC-MS/MS identification of α1 and α2 chains.

Protein	Accession no.	Score	Mass (Da)/PI	Peptide hits	Coverage (%)	Protein description
CSSCα1	XP_035626829.1	1996	137955/5.53	28	30%	Collagen alpha 1(I), (*Oncorhynchus keta*)
CSSCα2	XP_035651756.1	1219	127004/9.24	28	30%	Collagen alpha 2(I), (*Oncorhynchus keta*)
NTSCα1	BAL40987.1	527	138225/5.64	18	21%	Collagen alpha 1(I), (*Oreochromis niloticus*)
NTSCα2	BAL40988.1	906	126856/9.18	22	25%	Collagen alpha 2(I), (*Oreochromis niloticus*)
